# Controlled Human Infections As a Tool to Reduce Uncertainty in Clinical Vaccine Development

**DOI:** 10.3389/fmed.2018.00297

**Published:** 2018-10-29

**Authors:** Meta Roestenberg, Ingrid M. C. Kamerling, Saco J. de Visser

**Affiliations:** ^1^Department of Parasitology and Infectious Diseases, Leiden University Medical Center, Leiden, Netherlands; ^2^Centre for Human Drug Research, Leiden, Netherlands; ^3^Paul Janssen Futurelab, Leiden, Netherlands

**Keywords:** vaccine, malaria, product development (PD) process, clinical development, low-income access

## Abstract

Vaccines can be extremely cost-effective public health measures. Unfortunately the research and development (R&D) of novel vaccines is suffering from rising costs and declining success rates. Because many vaccines target low- and middle income markets (LMIC), output needs to be maintained at a constrained budget. In addition, scientific neglect and political uncertainty around reimbursement decisions make it an unattractive arena for private investors. The vaccine development pipeline for LMIC thus is in need for a different, sustainable, and cost-effective development model. In conventional vaccine development, objectives for every clinical development phase have been predefined. However, given the scarcity of resources, the most efficient clinical development path should identify vaccine candidates with the highest potential impact as soon as possible. We argue for a custom-made question-based development path based on the scientific questions, success probabilities and investments required. One question can be addressed by several studies and one study can provide partial answers to multiple questions. An example of a question-based approach is the implementation of a controlled human malaria infection model (CHMI). Malaria vaccine R&D faces major scientific challenges and has limited resources. Therefore, early preliminary efficacy data needs to be obtained in order to reallocate resources as efficiently as possible and reduce clinical development costs. To meet this demand, novel malaria vaccines are tested for efficacy in so-called CHMI trials in which small groups of healthy volunteers are vaccinated and subsequently infected with malaria. Early evaluation studies of critical questions, such as CHMI, are highly rewarding, since they prevent expenditures on projects that are unlikely to succeed. Each set of estimated probabilities and costs (combined with market value) will have its own optimal priority sequence of questions to address. Algorithms can be designed to determine the optimal order in which questions should be addressed. Experimental infections of healthy volunteers is an example of how a question-based approach to vaccine development can be implemented and has the potential to change the arena of clinical vaccine development.

## Introduction

Vaccines are one of the world's most important and cost-effective public health measures and have proven to generate vast socio-economic benefits (1). The elimination of smallpox and the near-elimination of polio as a consequence of global use of vaccines demonstrates the potential impact of these pharmaceuticals. As such, prophylactic vaccines have a unique niche in the pharmaceutical industry. Unfortunately, the rising development costs needed to maintain a constant output of new drugs over the past decades has affected the vaccine portfolio of major pharmaceutical companies.

Initially, investors perceived vaccine development to be riskier than other products, but this view has changed over the past years (2). Unfortunately, the overall probability of success of around 11% is not unlike any other pharmaceutical agent (2). Because the development of new vaccines is often complex, average development timelines are between 8 and 18.5 years, and estimated costs are substantial ($200 million to $900 million) (3, 4). Remarkable examples in this respect are the accelerated development of an Ebola vaccine and the long timelines in the development of an HIV vaccine. The risk of failure is particularly high in later stages of clinical development when vaccines are tested for their immunogenicity and protective efficacy in larger (target) populations (2, 4). Given the fierce competition, the fact that so-called “low-hanging fruits” have been picked, and in the aftermath of the global financial crisis, the vaccine development pipeline seems to encounter greater challenges than ever before (3).

## Challenges associated with vaccines for low- and middle-income markets

Because the global viral, bacterial, and parasitic infectious disease burden nowadays is primarily borne by low- and middle- income countries (LMIC), novel vaccines need to target LMIC markets. The limited financial capacity of such nations puts constraints on the vaccine market prices. Advanced scientific technologies in areas such as immunology, chemistry and molecular biology have accelerated vaccine development through, for example, increased understanding of population differences in vaccine efficacy (3) or identification of correlates of protection (4). While improving existing vaccines using incremental innovations can be done relatively fast, the remaining infectious diseases predominantly prevalent in LMIC require development of a novel category of vaccines, the foundation of which need to be laid by fundamental scientists. Whereas governments, non-governmental organizations and international donors play a central role in pre-clinical and early clinical development stages of vaccines, it is likely that private partners are eventually needed to successfully develop a vaccine for the (LMIC) market.

Predicting vaccine demand in the LMIC countries is difficult because the infrastructure needed to provide necessary epidemiological data and information on immunization coverage and wastage is sometimes lacking. In addition, vaccine uptake in the Global Alliance for Vaccines and Immunization (GAVI)-eligible countries may lag behind demand forecasts (3). The political uncertainty in reimbursement decisions and the public pressure to reduce prices limits the enthusiasm for private investors to enter this arena. The current procurement systems and strong downward price pressures further increase the uncertainty of recovering costs of development and goods (5). Lastly, differential pricing, despite its proven public health success, has been jeopardized by so-called “external price referencing,” whereby high-income countries seek to benefit from the lower prices offered to countries with weaker economic profiles (3).

Given the current vaccine development landscape, the market-driven business model will need to be revisited in order to provide a feasible, sustainable, and cost- effective structure for a global population.

## Improving the business case for vaccine development

Global recognition of the challenges in vaccine development for LMIC has increased the support of donors for vaccine research, particularly from the Bill and Melinda Gates Foundation (6). These and other direct grants and investment in product development partnerships (PDPs) have enabled a more active participation of public partners later in the clinical development pipeline. Public investments reduce R&D costs and improve the business case for private partners. Developing country manufacturers have been able to reduce the production costs of several vaccines, substantially increasing vaccine cost-effectiveness and ultimately the population reached. However, the involvement of large pharmaceutical companies, including developing country manufacturers, is substantially higher at later stages of clinical development, reflecting the preference of private investors for lower risk development. Forty percent (40%) of the R&D efforts currently invested in neglected diseases is conducted through product development partnerships (PDPs) (3). However, also public donors increasingly demand success given hundreds of vaccine candidates in development globally (7). Considering the resource constraints, there is a growing need to rationally identify the approaches that are most likely to succeed and then prioritize among these candidates (8).

Alternatively, market-based “pull” mechanisms—where donors stimulate demand for new technologies through purchase commitments and volume guarantees—incentivize vaccine research and development by fuelling the business case from the revenue side. Similarly, effort is put into defining target product profiles for new vaccines early in the development process, aiming to reduce risks of late failures by being explicit about the requirements for novel category vaccines (5). However, “pull” mechanisms work best when the concept of a new vaccine is proven and the intrinsic development risks are reduced to acceptable, technical risks (3).

In conclusion, managing costs and risks of vaccines for LMIC is challenging. Providing early proof-of-concept of these vaccines is essential in order to prioritize and ensure donor enthusiasm, raise funds from private investors or fuel collaborations with companies. Obviously, any product will have intrinsic development risks which are technical in nature. However, additional uncertainty may be introduced by the lack of scientific insights which need to be valued within the vaccine portfolio. Unfortunately, investment decisions on R&D projects in life sciences are frequently based on Net Present Value (NPV) calculations that depend heavily on assumptions of technical risks, costs and future profits while scientific considerations are not taken into account. NPV analysis just indicates that such evaluation studies cost time and money and disregards the increases in knowledge which is obtained when scientific questions are adequately addressed. Particularly in vaccine development for LMIC scientific advances are fundamental to game-changing novel technologies.

## Question based clinical vaccine development

Classically, the clinical development program of a vaccine is divided in four phases:

- Phase 1: Research using small groups of healthy volunteers. Traditionally, this phase mainly focuses on vaccine safety, may explore its immunogenicity, and targets to finding a dose where the level of tolerance is acceptable. In general, this phase takes about 1–2 years.
- Phase 2: Clinical trials are larger and the first proof for immunogenicity is established. More characteristics of the vaccine are determined and a safe and well-tolerated dose is determined where the drug is immunogenic.
- Phase 3: The potential new vaccine is tested on thousands of patients in an endemic setting to investigate its safety in more detail. Furthermore, the efficacy of the vaccine at the determined dose is determined. Further research is conducted to investigate possible side effects after long-term treatment and development of the drug for different indications is investigated.
- Phase 4: The registered and introduced vaccine is monitored closely to examine the occurrence of unexpected side-effects and interactions with other vaccines.

**Figure 1:**
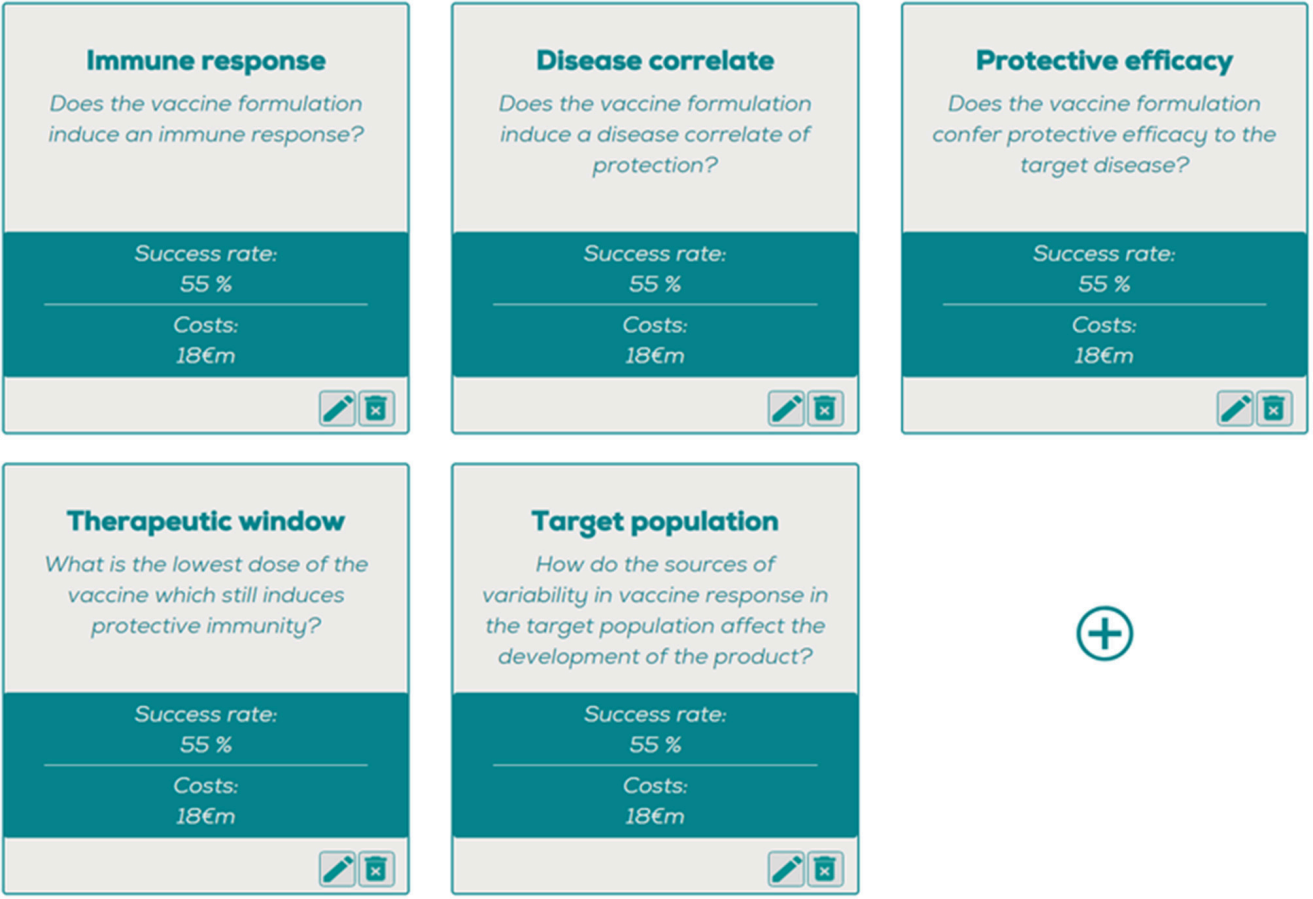
The question optimizing tool, where estimated costs and probabil ities of success can be entered per question, generating a question-based decision tree with the most optimal order of questions. https://www.pauljanssenfuturelab.eu/our-tools/.

The description of these phases is typically process oriented and contains very little information about which scientific aspects are actually covered during the clinical development. Alternatively, the clinical vaccine development pipeline can be centered around key scientific questions which need to be addressed in the most optimal order applicable to the individual investigational vaccine (Figure 1). Examples of such questions are:

- *Does the vaccine formulation induce an immune response (“****Immunogenicity”****)?*

This main generic question contains several issues that need to be addressed such as the route and site of vaccine administration. Not only the immunogenicity of the vaccine antigen, but also any possible adjuvants, vectors or conjugates could be included in answering this question.

- *Does the vaccine formulation induce a disease correlate of protection (“****Disease correlate***”*)?*

Answering this question includes the demonstration of the immunological mechanism of action for the investigational vaccine. As stated previously, R&D investments into mechanisms of disease and correlates of protection are essential to help answer this question and as such the associated development risks in this area may vary considerably. Addressing this question may be very instrumental in addressing the other questions also. In diseases where a correlate of protection is lacking, e.g., malaria or HIV, protective efficacy trials bear substantial risks which have impeded vaccine development.

- *Does the vaccine formulation confer protective efficacy to the target disease (“****Protective efficacy***”*)?*

This question reflects the need to establish beneficial effects on the incidence or prevalence disease but also the alteration of other physiological systems resulting in clinical side effects. Depending on the infection incidence, these trials can be particularly large in vaccine development in order to achieve sufficient power to detect efficacy.

- *What is the lowest dose and number of doses at which the vaccine which still induces protective immunity (“****Therapeutic window***”*)?*

The therapeutic window of each investigational vaccine needs to be established in order to select the optimal dose that is clinically optimally efficacious at well tolerated levels. This question includes important sub-questions: how many vaccine doses need to be given at what interval? Can vaccine formulation decrease the need for booster doses?

- *How do the sources of variability in vaccine response in the target population affect the development of the product (“****Target population***”*)?*

This question should include: Are there any specific factors in the target population that may affect immunogenicity? Particularly in vaccine development for LMIC this can be a significant hurdle given the generally decreased efficacy of vaccines in resource-poor settings (9). Co-infections, previous exposure to the target disease or malnutrition may be factors which hamper immunogenicity in the target population (10, 11).

**Figure 2:**
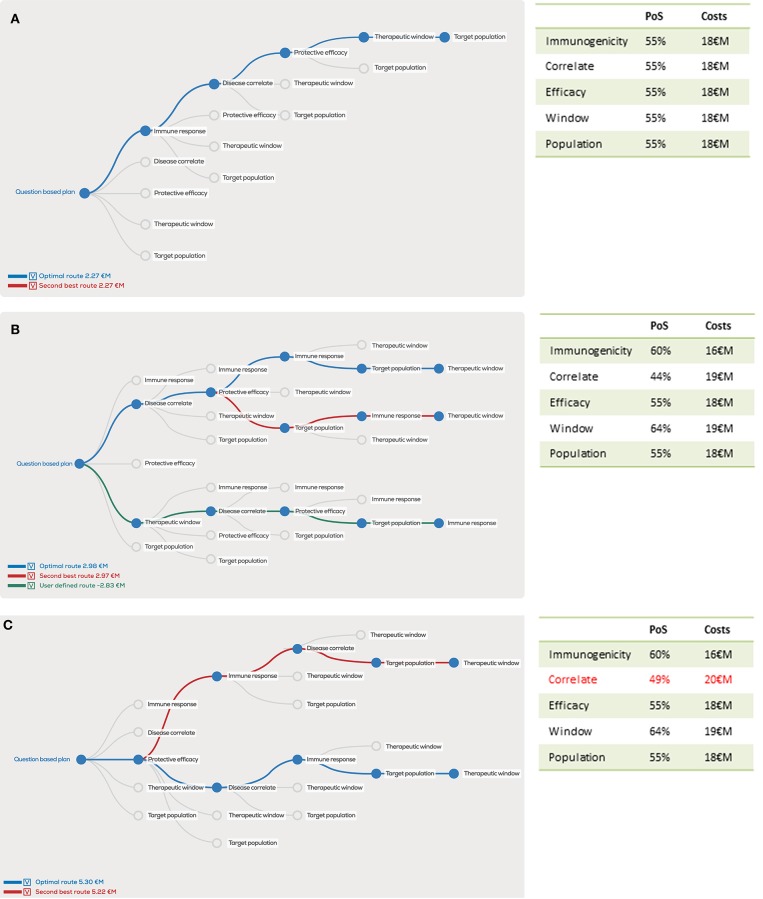
Decision tree showing the optimal order of questions to generate the highest project value, given estimated probability of success (PoS) and costs (million euro, €M) per scientific question. In the case PoS and costs for every question are equal, the order does not matter for the project value **(A)**, however unequal distributions will clearly show different project values for the optimal order in blue, second best order in red and user defined order in green **(B)**. A 1 €M additional investment in “correlates” has a major effect on the optimal order and project value **(C)**.

The abovementioned questions can be ranked based on their risk profile and examined in different sequence orders. For example, if we examine the clinical development path for a hypothetical malaria vaccine using overall probability of success of 5%, development costs of 90 €M and revenues of 800 €M. Equal distribution of the risks and costs over the five proposed questions would amount to a 55% probability of success and 18 M€ costs for each question. The resulting question-based decision tree (Figure 2A) reflects the true scientific risks and uncertainties that are faced in the development of an individual vaccine based on estimations of risks associated with the postulated questions.

Because it is unlikely that all five questions would contribute evenly to the overall risks, we now unevenly distribute risks and costs in our example (Figure 2B). Despite similar overall costs and risks, the project value can vary substantially depending on the distribution of question-associated risks and costs. Similarly, the order of questions strongly determines the project value as illustrated by the dramatic drop in project value if the user-defined “Therapeutic window” is the first question to be addressed with these input variables. In this case, the overall project value actually drops below zero, implying that risks and investments do not outweigh the potential revenues.

Furthermore, the question-based approach illustrates how the project value can increase by performing an additional early phase evaluation study that helps to adequately answer a critical question. If an additional 1 M€ costs and a 5% increased probability of successfully answering the “Disease correlate” question is added, the overall project value increases by more than 50% (Figure 2C), despite the fact that the overall probability of success increases with only 0.17–5.17% and overall costs increase by 1–91 M€.

By defining the costs and probabilities of success and constructing the decision tree for every new vaccine, the bottleneck in the development of each individual vaccine will be identified. Presumably for most vaccines the “Disease correlate” and “Protective efficacy” questions will have the lowest probability of success. As explained earlier, the increasing recognition of this critical bottleneck in vaccine development has led to a demand for proof-of-concept clinical trials showing early efficacy for the candidate vaccine technology. In the next section, we will take the example of malaria vaccine development to illustrate how question-based product development has changed malaria vaccine development and fostered novel technology development in this field.

## The example of malaria vaccine development and the role of controlled human malaria infections

Despite the fact that half the world's population is at risk for malaria with an extremely high global burden of 200 million malaria cases annually and nearly 500,000 deaths among children, the vast majority of the burden is borne by resource-poor countries in Sub-Saharan Africa (12). Most malaria deaths are caused by one microbial species: the protozoan *Plasmodium falciparum (Pf)*. This organism displays extracellular and intracellular developmental stages and transforms within the human host, creating a challenge for the immune system and therefore also a scientific challenge for malaria vaccine development. Because there are no prior registered vaccines for parasitic diseases, malaria vaccine development cannot draw on experience from other disease areas. In addition, despite decades of vaccine research, there are no correlates of protection for *Pf* malaria. Lastly, *Pf* strains worldwide display considerable genetic variability which may affect local vaccine efficacy. As a consequence, the probability of successful market entry for malaria vaccines is extremely low even after 7–8 years of clinical development (4) and the investment into malaria vaccines is extremely small as compared to other disease areas such as influenza or HIV/AIDS (4). A radical approach to tackle R&D uncertainty and boost scientific advances is thus urgently needed.

Initially developed as a treatment for neurosyphilis before the discovery of penicillin, the methods for experimental infection of volunteers with malaria have been adapted as a tool to serve the malaria vaccine pipeline (13). The human infection model was standardized by means of (automated) parasite cultures and laboratory-bred mosquito colonies (14, 15). Nowadays, both the blood-stage as well as the mosquito stage of the Pf parasite can be GMP manufactured and injected for this purpose (16, 17). The model is known as “controlled human malaria infection (CHMI)” to stress the importance of the standardization and the highly controlled follow-up which ensures that severe malaria does not occur and participants are treated as early as possible.

Within the vaccine development pipeline, CHMI has been increasingly used to address the “Disease correlate” question in the absence of a known correlate of protection. Despite the fact that a formal validation was never done, CHMI trials are nowadays widely accepted as a critical step in the clinical development path. Using the CHMI model, one vaccine—GlaxoSmithKline Mosquirix—has received marked authorization in 2016 following a proof-of-concept clinical trial more than 10 years earlier (18). An implementation pilot to evaluate its use in the field is currently ongoing. The product development partnership “Malaria Vaccine Initiative,” has very successfully advanced this vaccine for the public sector and simultaneously managed global access and IP issues with commercial partners (5). CHMI has also fostered unconventional approaches to vaccine development such as the live-attenuated malaria vaccine PfSPZ Vaccine which is entering phase 3 clinical trials, led by the US biotech company Sanaria (17). Furthermore, disappointing CHMI results have stopped the development other candidate malaria vaccines (19).

The CHMI trials are thus a prime example how questions with the lowest probability of success can be addressed in early clinical development. Because of their invasive nature and the requirement for healthy volunteers, which seemingly contradict the principle of “primum non nocere,” CHMI trials initially raised ethical debate but nowadays have gained acceptance through demonstrating their accelerating scientific potential and the fact that CHMIs have been safely performed in >3,000 volunteers worldwide (20).

Following the example of malaria, the experimental infection of volunteers are also being used as a tool for the development of novel products in other infectious disease areas such as influenza, rhinovirus or cholera (21). In exceptional cases, such as the cholera vaccine VaxChora, this trial was the basis for registration of a novel vaccine (21). However, the position of CHI trials within the vaccine development pipeline and their value in mitigating the development risk is highly dependent on its scientific validity. Therefore, attention should be paid to the scientific details of the CHI trial setup, in particular to the proposed vaccine mechanism of action, the vaccine target population and the position of the CHI model within the vaccine product pipeline. Specific points to be considered are outlined below.

## Restrictions and solutions for CHI models

### Mechanism of action

Vaccines consist of one or multiple antigens and as such target prevention of disease, infection or colonization. Because CHI trials are often designed as preliminary experiments which only include a very small group of volunteers, endpoints should be selected based on their power to discriminate vaccine effects. Preferably, these trials target a relevant (clinical) endpoint, addressing a claim in the vaccine target product profile, such as diarrhea in the case of cholera CHI (22) or fever in the case of typhoid CHI (23). Alternatively, an intermediate (microbiological) endpoint may be selected, such as viremia in the dengue model (24) or parasitemia in the malaria model. However, for malaria, infection does not always parallel disease particularly in endemic areas (25). Depending on the target disease the CHI endpoint as such may be a surrogate to clinical endpoint and will need to be validated in epidemiological studies or in later field trials (Table 1).

**Table 1:** Examples of potential restrictions and solutions for CHI models within the vaccine product pipeline.

**Topic**	**Restriction**	**Solutions**
Mechanism of action	Model endpoint does not reflect vaccine efficacy endpoint	Define endpoints to balance clinical relevance and feasibility in small groups. Validation of endpoints in epidemiological studies.
	Challenge strain does not express vaccine antigen, or correct strain not available	Design of fit-for-purpose challenge strains or models
Model validation	Model population does not reflect target population	Transfer of CHI trials to endemic areas and susceptible populations
	Challenge strain does not reflect circulating field strains	Increase portfolio of challenge strains to reflect natural infections
Position of CHI in product development pipeline	False negative result in CHI trial leads to no-go decision for further development of potentially valuable product	Clear definition of the research question which the model addresses
	Acceptance of CHI data in registration dossiers	Early involvement of regulators

In addition, the vaccine antigen(s) should be sufficiently present in the CHI model to measure protective effects. For example, in the malaria case, a liver-stage malaria vaccine can be tested for its preliminary efficacy by a mosquito-bite CHMI. However, for blood stage malaria vaccines, this model is less suitable because blood stage parasitemia will be limited to only a very short (3–4 day) timeframe in a mosquito bite CHMI (26). Therefore, the CHMI model was adapted to accommodate a longer phase of blood stage parasitemia. This blood stage CHMI has the possibility to detect small alterations in blood stage development of malaria parasites (16).

### Model validation

Generally, CHI trials are performed in healthy adult volunteers which do not necessarily reflect the target population, which is typically much more heterogeneous. In order to overcome these differences, CHI trials can also be performed in more susceptible (target) populations such as COPD patients for rhinovirus (27) or malaria infections in Sub-Saharan Africa (28). Interestingly, controlled human malaria infections in non-endemic high-income settings have a much higher clinical attack rate and parasitemia as compared to rural African populations. In the latter population parasitemia is much more variable, and clinical disease may be lacking despite parasitemia (25). It is plausible that this reflects different immunological responses, which may be unraveled in the future.

Given the invasive nature of the CHI trial, regulatory authorities will often demand strictly controlled production of challenge material. In addition to the fact that such processes may be difficult, expensive and time-consuming, the process itself may render the challenge material less representative of microbes circulating in the field. For example, passage of virus strains through well-characterized cell lines to produce Good Manufacturing Practice compliant strains, will unequivocally alter the genetic makeup of the virus. Alternatively, the well-characterized laboratory strains such as the Quailes strain used for typhoid CHI (23) or the NF54/3D7 strain for *Pf* CHI (20) may not reflect the heterogeneity of field strains which impacts the scientific value of the model to predict field efficacy.

### Position of CHI in the product development pipeline

Depending on the scientific and clinical details of the CHI model mentioned above, the CHI model can be used as a tool to answer one or multiple questions in the product development pipeline. Determining the most optimal order in which these questions should be addressed, will aid in positioning the CHI model in the pipeline. Because CHI models often address high-risk clinical development questions, they are optimally performed early in clinical development. Previously, this has led to hesitancy by vaccine developers, who fear that negative results will result in a “no-go” decision for a vaccine which may actually be efficacious in later trials. However, as in any other model, the results of the model system should be valued for its merits within the scientific question which it addresses. For example, a CHMI trial may enable identification of an immunological correlate of protection in a trial which shows only partial efficacy. The correlate will de-risk other clinical development questions. Depending on the outcome of the trial, these need to be reassessed and risks adjusted to come up with the then optimal order based on the required investments and updated probability of success.

Increasing familiarity of CHI models by regulators as well as vaccine developers increases acceptance of these trials as part of the regulatory package which is submitted for licensure. However, given the restrictions of CHI models, continuous education of regulators, and vaccine developers to increase the scientific understanding of these models is essential to ensure that data from these trials are interpreted appropriately. In the end, regulation follows science, not the other way around. The malaria example shows how a well-designed CHI trial early in clinical development can dramatically improve the business case of the experimental vaccine and ultimately lead to registration of a LMIC vaccine with global impact.

## Conclusions

In conclusion, global vaccine development faces major challenges, particularly for LMIC settings. Initiatives to improve the business case for vaccine development including so-called “pull” mechanisms to ensure pricing and guarantee demand are needed but will not provide the ultimate solution. A question-based clinical development approach can provide the insights on critical steps in the development path for novel vaccines and will help display the priorities within the program. Controlled human infections, if well designed, are an excellent example of how question based product development has led to adjustments to the product development pipeline and the way to prioritize vaccine candidates, accelerate novel vaccines, reallocate resources and foster novel technologies. During development, the estimations in the question based approach require constant evaluation and adjustment in order to keep development on the optimal path. Ultimately, this approach has the potential to more quickly improve global health and hopefully increase the appetite for private investors to enter the arena of clinical vaccine development and work together with public funders to target low income markets despite small profit margins. In addition, it will foster scientific advances which are needed to turn the tide on the development of vaccines for neglected infections of global importance, increase enthusiasm for public investments and the public pressure needed to stimulate societal corporate responsibility.

## Author contributions

MR drafted the manuscript. IK and SdV reviewed and finalized the manuscript.

### Conflict of interest statement

The authors declare that the research was conducted in the absence of any commercial or financial relationships that could be construed as a potential conflict of interest.
