# Corrigendum: Controlled Human Infections As a Tool to Reduce Uncertainty in Clinical Vaccine Development

**DOI:** 10.3389/fmed.2019.00023

**Published:** 2019-02-14

**Authors:** Meta Roestenberg, Ingrid M. C. Kamerling, Saco J. de Visser

**Affiliations:** ^1^Department of Parasitology and Infectious Diseases, Leiden University Medical Center, Leiden, Netherlands; ^2^Centre for Human Drug Research, Leiden, Netherlands; ^3^Paul Janssen Futurelab, Leiden, Netherlands

**Keywords:** vaccine, malaria, product development (PD) process, clinical development, low-income access

In the original article, there was a mistake in “Figure 2C” as published. In the figure legend, the incorrect number was used to indicate the second-best route and should have been “5.22 €M” instead of “5.97 €M”. The corrected Figure 2C appears below.

**Figure 2:**
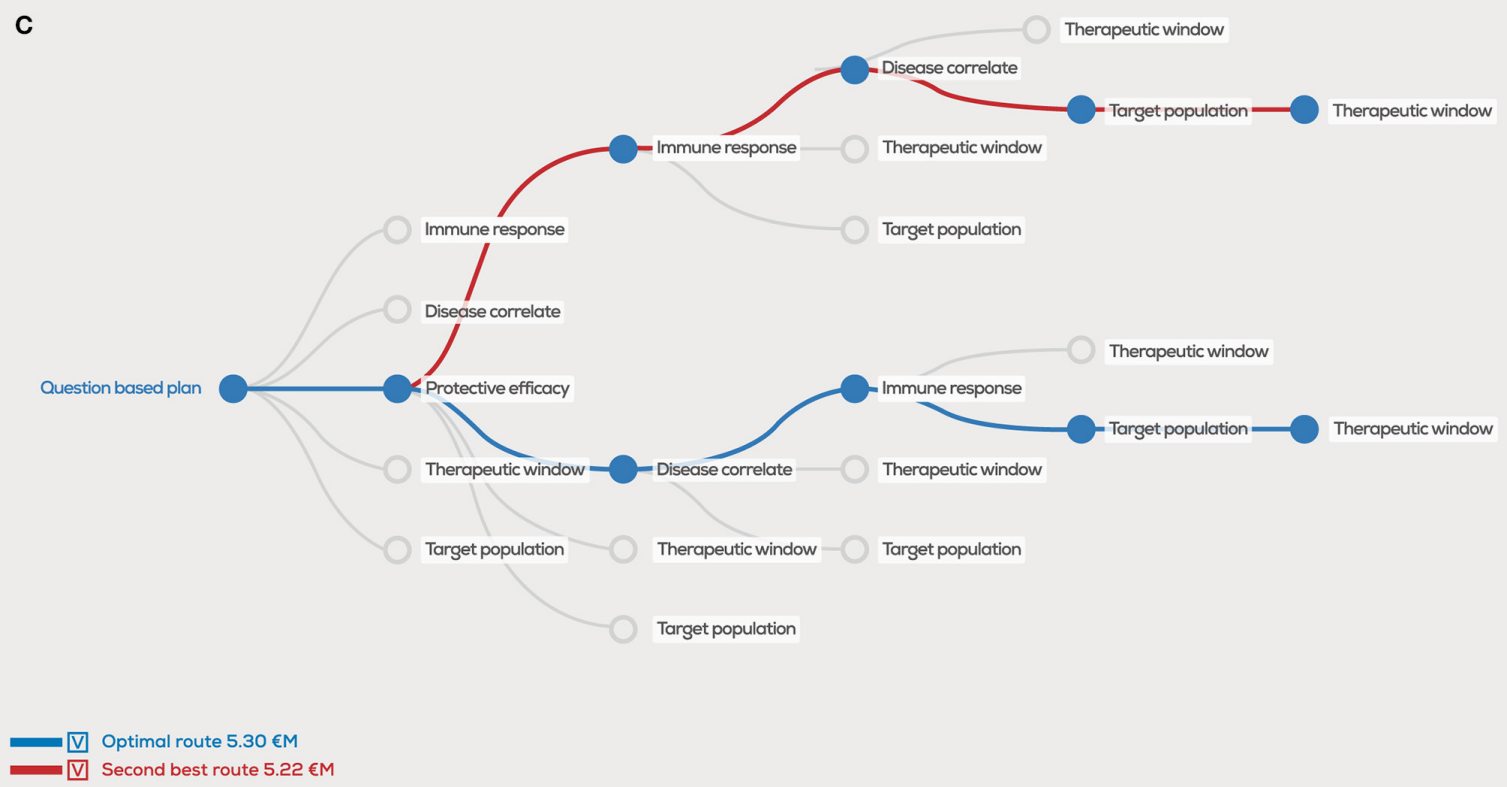
Decision tree showing the optimal order of questions to generate the highest project value, given estimated probability of success (PoS) and costs (million euro, €M) per scientific question. In the case PoS and costs for every question are equal, the order does not matter for the project value **(A)**, however unequal distributions will clearly show different project values for the optimal order in blue, second best order in red and user defined order in green **(B)**. A 1 €M additional investment in “correlates” has a major effect on the optimal order and project value **(C)**.

The authors apologize for this error and state that this does not change the scientific conclusions of the article in any way. The original article has been updated.

